# An Experimental Intervention Study Assessing the Impact of a Thin Silicone Gel Surface Overlay on Interface Pressure

**DOI:** 10.1155/2020/3246531

**Published:** 2020-11-24

**Authors:** Seth Kwadjo Angmorterh, Andrew England, Sonia Aboagye, Eric Kwasi Ofori, Peter Hogg

**Affiliations:** ^1^Department of Medical Imaging, School of Allied Health Sciences, University of Health and Allied Sciences (UHAS), Ho, Ghana; ^2^School of Allied Health Professions, Keele University, Staffordshire, Newcastle, UK; ^3^Department of Speech, Language & Hearing Sciences, School of Allied Health Sciences, University of Health and Allied Sciences (UHAS), Ho, Ghana; ^4^Directorate of Radiography, School of Healthcare Sciences, Allerton Building, University of Salford, Salford—Greater Manchester, UK

## Abstract

**Introduction:**

The incidence of pressure ulcers (PUs) presents a substantial threat to patients, especially geriatric patients, those with restricted mobility, and patients suffering from chronic diseases such as cancer. PUs creates a huge financial burden on healthcare authorities and patients, costing billions to treat and manage. Radiography and radiotherapy patients may experience medical device related (MDR) PUs and studies have shown that high interface pressure (IP) values exist for the head when placed on an X-ray table without a mattress. These high IP values pose a PU risk to patients, especially those accessing prolonged radiography/radiology and radiotherapy procedures. The current study assessed the impact on IP values for the head from using a thin silicone gel surface overlay during radiographic procedures and identified whether this reduced the risk of PUs.

**Materials and Methods:**

A calibrated XSENSOR pressure mat was used to measure IP for the head on an X-ray table with and without a thin silicone gel surface overlay. Prior to pressure mapping, the silicone gel surface overlay was assessed for its impact on radiation attenuation and image quality.

**Results:**

Study participants were 14 males (70%) and six females (30%), with an age range of 25–53 years (mean = 34.4 ± 7.0). Paired-samples *t*-test results indicated that there was a statistically significant decrease in the mean IP for the head on the X-ray table without the silicone gel surface overlay (mean = 83.9 ± 8.2 in mmHg) and the X-ray table with the gel surface overlay (mean = 62.4 ± 6.1 in mmHg), *p* ≤ 0.001. Paired-samples *t*-test results indicated that there was a statistically significant decrease in the mean peak pressure index (PPI) for the head on the X-ray table without the silicone gel surface overlay (mean = 205.1 ± 28.2 in mmHg) and the X-ray table with the gel surface overlay (mean = 159.8 ± 26.8 in mmHg), *p* ≤ 0.001.

**Conclusions:**

The use of a thin silicone gel surface overlay could reduce IP risk for the head by approximately 25%. The reduction in IP risk could have a significant impact in reducing the risk of developing a PU. To ensure maximum benefit, the silicone gel surface overlay should be evaluated to address the specific needs within radiography and radiotherapy planning and treatment settings.

## 1. Introduction

Pressure ulcers (PUs) are wounds to the skin and underlying tissue caused by sustained pressure on the skin. They can develop within a short space of time or over days. Geriatric patients, those with restricted mobility, and patients suffering from chronic diseases such as cancer are at greater risk of developing a PU [1, 2]. PUs are a common problem in healthcare and significant effort and international attention has been directed towards reducing their incidence. However, rates continue to rise, resulting in increasing numbers of PU sufferers worldwide [3, 4]. Prevalence of PUs across hospital settings and nursing homes in the United Kingdom (UK), Canada, and the United States of America (USA) is 4.7%, 36.8%, and 12.3%, respectively, [5–7]. PUs place a huge financial burden on healthcare authorities, costing billions to treat and manage [8–10]. They also lead to negative physical and psychological effects on patients, thereby reducing their quality of life [11, 12]. Due to the harmful effects of PUs, the National Institute for Health and Care Excellence (NICE) of the United Kingdom (UK) has recommended that rigorous scientific research should be conducted into the aetiology and prevention of PUs to help prevent or minimise their incidence [13].

In radiography and radiotherapy, patients are likely to be exposed to medical device related (MDR) PUs. These are localised injuries to the skin and/or underlying tissue because of sustained pressure from a medical or therapeutic device [14]. MDRs usually appear visually on the superficial layer of the skin and take the shape of the device [15, 16]. In radiography, because of the need to minimise error and produce diagnostically acceptable images, patients are usually transferred onto medical imaging surfaces prior to a procedure [17]. These hard surfaces often have a thin radiolucent mattress on their surface, to aid patient comfort. During radiographic procedures, a pillow may be used, which has valuable consequences for PU minimisation. However not all cases permit a pillow to be used. Furthermore, in some countries such as Portugal and Ghana, diagnostic radiography procedures are typically conducted on hard carbon fibre X-ray tables. By contrast, in radiotherapy, it is important to maintain reproducibility of patient position during planning and treatment, so patients are usually positioned on hard couch surfaces again with no mattress [18]. It is essential that the daily radiotherapy treatment position is the same as that in planning to ensure accuracy of the radiotherapy procedure [19]. Lying on hard imaging and radiotherapy treatment surfaces with no mattress could be harmful to at-risk populations such as elderly patients and those suffering from cancer because of their fragile skin [20]. Confounding this is the fact that some of these procedures take a very long time to complete. For example, cranial stereotactic radiotherapy takes between 40 and 60 minutes depending on the clinical history of the patient [21]. Cervical vertebroplasty, an interventional radiography procedure, takes over an hour to complete and sometimes longer when several cervical fractures are present [22]. Another confounding factor is that patients are intentionally immobilised to minimise image artefacts during the procedure. Immobilisation is harsher in radiotherapy because patient positioning during treatment needs to be assured. For example, the use of immobilisation devices such as full head masks helps to reduce positioning errors but limits patient motion. These are necessary to minimise misdirection of prescribed radiation doses [19]. All these factors could contribute to high interface pressure (IP) between the head and the radiography/radiotherapy surface.

Interface pressure (IP), defined as the pressure between the human body and a supporting surface, plays a crucial role in skin damage [23, 24]. Seminal works have shown that IP greater than 47 mmHg sustained for a period longer between 30 minutes is most likely to compromise blood circulation and may cause tissue ischaemia, which may lead to PUs [25–29]. PUs are prone to occurring at the head (occiput), sacrum, and heels, due to the prominent bony features found at these anatomical sites [30, 31]. A study conducted by Justham et al. [32] indicated that there is the potential of high IP on medical imaging and radiotherapy surfaces. Angmorterh et al. [33] followed up on this study and more recently confirmed that high IP values do exist on X-ray tables without mattresses. The high IP values pose a PU risk to patients and could increase the risk of developing PUs in patients accessing prolonged radiography/radiology and radiotherapy procedures. High IP risk could have a more severe negative impact among geriatric patients and those suffering from chronic diseases such as cancer due to the poor collagen and elastin content in their skin and the presence of comorbidities among these patient populations [34]. The study by Angmorterh et al. [33] also found that lying on an X-ray table without a mattress can be very uncomfortable and, in some cases, patients may experience pain centred in the head region. Such discomfort could have negative implications on patient management as research has highlighted a link between patient comfort and the accuracy of radiotherapy procedures [35].

It is common for patients not to be given pillows during radiographic and/or radiotherapy procedures as the pillow could induce diagnostic and radiotherapy planning and treatment errors. In some countries, for example, Ghana, fluoroscopic X-ray machines do not use mattresses. Patients undergoing fluoroscopic procedures such as cervical vertebroplasty are required to lie on hard rigid fluoroscopic X-ray surfaces for the duration of the procedure. It must be stated that patients' heads are not supported on pillows during cervical vertebroplasty due to the possibility that the pillow might elevate the head above the level of the cervical spine, thereby putting pressure on the already distressed cervical spine. These may both increase the pain in the cervical spine, as well as cement leaks within the vertebrae [36]. Additionally, the proximity of the cervical vertebrae to the head demands that the head is not supported on pillows because the use of pillows could produce artefacts, which might affect the diagnostic quality of the fluoroscopic image. It is a common practice in radiography and radiotherapy that any anatomical area which to be irradiated and its immediate surroundings are kept free of foreign materials [17]. However, the absence of a pillow or any form of cushioning at the head, combined with rigid immobilisation, could induce tissue damage because the head will be in direct contact with a rigid fluoroscopic surface for prolonged period of time. It is therefore important to explore ways of minimising the high IP risks identified for the head when using X-ray tables without mattresses. This empirical intervention study aimed to assess the results of using a thin silicone gel surface overlay as an intervention to minimise the high IP risk for the head. The outcome of this study may help inform measures to reduce the incidence of PUs among patients accessing prolonged radiography and radiotherapy planning and treatment procedures.

## 2. Materials and Methods

### 2.1. Study Design, Setting, and Ethical Considerations

This was a quantitative experimental study, conducted at the medical imaging facility of the University of Salford (UoS) in Manchester, UK. The study was approved by the UoS College of Health and Social Care Ethics Committee (HSCR 15–141).

### 2.2. Pressure Redistributing Surface Overlays

Pressure redistributing surface overlays are characterised by design, by materials in the finished product, and as dynamic (alternating) or static (constant). The primary aim of pressure redistributing support surfaces such as mattresses, surface overlays, and cushions is to relieve IP so as to provide some level of cushioning to high risk parts of the body and distribute the IP more evenly. The two main types of pressure redistributing surface overlays—alternating and static—provide different functions [37]. Alternating pressure redistribution surface overlays cause periodic high and low movement. However, they cannot be applied in radiography or radiotherapy planning and treatment procedures because of the risk of errors and subsequent negative impact on patient management. For the purposes of this intervention study, a range of static pressure surface overlays were scrutinized for selection. Foams and air-filled surface overlays are not applicable in radiotherapy planning and treatment procedures because they have the tendency to squeeze and sometimes collapse under patient weight. This can induce movement during procedures; hence, they were not used in this study. Following extensive searching and contact with clinicians, tissue viability nurses, occupational therapists, manufacturers, and distributors of pressure redistribution surface overlays, five static pressure redistribution surface overlays were identified and procured. The physical characteristics of the surface overlays are detailed in Table 1.

### 2.3. Radiation Tests

Radiation tests were conducted on the five pressure redistribution surface overlays listed in Table 1. The main aim of these tests was to determine the one with the least impact on radiation dose attenuation and image quality and apply it during interventional radiography and radiotherapy planning and treatment procedures. The radiation tests were conducted in three stages. Firstly, a dosimetry test was conducted to assess the impact of each surface on radiation attenuation. Secondly, an assessment was made of the impact of each surface overlay on image quality. Thirdly, computed tomography (CT) scanning of each surface overlay was conducted to provide a detailed internal three-dimensional visualisation of each surface overlay. The information from the CT images was used to calculate the Hounsfield unit, hence, the linear attenuation coefficient of each surface overlay. The attenuation coefficient is a measure of how easily a surface overlay can be penetrated by an incident X-ray beam and describes the fraction of a beam of X-ray that is absorbed or scattered per unit thickness of the surface overlay.

#### 2.3.1. Dosimetry Test

The X2 R/F dosimeter (RaySafe, UK) is a modern piece of equipment fitted with high sensor technology that ensures accurate measurement of radiation dose. It was used to assess the impact of each surface overlay on radiation dose attenuation. The dosimeter has a dose range of 40–150 kVp and can detect dosages ranging from 1 nGy to 9999 Gy, with an accuracy of ±5% of calibrated values. The X2 R/F dosimeter has the ability to measure dose rate, peak kilovoltage (kVp), half-value layer (HVL), total filtration, exposure time, pulses, pulse rate, and dose/pulse in one exposure.

The method used for the dosimetry test involved placing the X2 R/F dosimeter on an X-ray table. The radiation field was tightly collimated to the edges of the dosimeter. Using a standard 100 cm source to image-receptor distance (SID), three exposures were made at both high kilovoltage (kV) (75 kV, 2 mAs) and low kV (50 kV, 2 mAs) with a fine focal spot. The mean recorded dose for high and low kVs was 37.28 and 10.72 mGy, respectively. These values served as the control. To assess the impact of each surface overlay on radiation dose attenuation, each surface overlay was placed on the dosimeter. Three exposures were made using the same exposure parameters as the control. The mean recorded dose for each surface overlay and the percentage difference from the control at low and high kVs is reported in Table 2.

To objectively determine the amount of radiation incident on the detector when the various pressure redistribution surface overlays were used, the exposure index (EI) and the deviation index (DI) of each of the X-ray images with the hand phantom were assessed. The EI is an international standard that measures the amount of radiation exposure on a digital image receptor [38]. A target exposure index (TI) value is a constant value set by the manufacturer for specific anatomical parts [39]. The TI value for the X-ray machine used for the study was 250 *μ*Gy. The deviation index (DI) calculates the difference between a desired TI and the actual exposure [39]. The EI and the DI for the exposures on the surface overlays are indicated in Table 3.

#### 2.3.2. Image Quality Assessment

The second part of the radiation tests involved assessing the impact of each surface overlay on radiographic image quality. Each pressure redistribution surface overlay was placed on a 17 × 14 inch Aero digital radiography (DR) cassette (Konica Minolta, Tokyo, Japan). Following this, they were exposed to radiation at high kV (120 kV, 1.2 mAs) and low kV (60 kV, 1.2 mAs) using fine focus. A 23 cm long adult hand anthropomorphic phantom weighing 0.79 kg was placed in the middle of the surface overlay on the cassette. The radiation field was collimated to an area of 20 × 25 cm to conform with clinical standards. The same exposure factors at both high and low kV were repeated. These exposure parameters are consistent with exposure protocols for the hand in clinical settings. In all, four exposures were taken for each surface overlay-two with the hand phantom placed on the surface overlay at high and low kVs and another two with only the surface overlay placed on the cassette, also at high and low kVs. The images were obtained and processed on AeroDR system (Konica Minolta, Inc.) and its workstation. The acquired radiographic images are presented in Table 4.

#### 2.3.3. Attenuation Coefficient Determination

The third and final stage of the radiation tests involved assessing the density of the various surface overlays to evaluate their Hounsfield unit (HU) measurements and determine the linear attenuation coefficients of the surface overlays. Each surface overlay was scanned using a Toshiba Aquilion 16 slice multidetector CT scanner. To calculate the mean HU of each surface overlay, an area corresponding to a number of pixels was chosen depending on the length and thickness of the surface overlay. For example, when calculating the HU for the silicone gel flat pad (surface 2), 12 areas of 0.2 cm^2^ (averaging 40 pixels) were chosen. The HU for each area was calculated and the mean HU for the entire silicone gel surface overlay was also calculated. The procedure was replicated to calculate the HU for the other surface overlays. The recorded mean HU and standard deviation (SD) of the surface overlays are presented in Table 5.

Based on the findings from the radiation tests, the silicone gel flat pad (surface overlay 2) was chosen as an intervention. It produced no artefact on the resultant radiographic image and had minimal impact on radiation dose attenuation due to a reasonably low linear attenuation coefficient. It also consists of fairly homogenous internal structures making it applicable for radiography and radiotherapy planning and treatment procedures.

### 2.4. Sampling for Pressure Mapping

A priori power analysis indicated that 20 volunteers would be needed for the research (effect size [0.67], power [0.80], alpha [0.05], and two-tailed repeated measures paired-samples *t*-tests). Effect size, power, and alpha value were determined from the study conducted by Angmorterh et al. [33]. A disproportionate stratified random sampling method was used to recruit 20 students and staff from the UoS. This sampling method was chosen to enable recruitment of volunteers with different characteristics. Inclusion criteria were healthy people aged 18 years and older. Exclusion criterion was volunteers who were >250 kg in weight. These are based on the limitations of the XSENSOR mat. Participation in the study was voluntary.

### 2.5. Data Collection Instrument

A calibrated XSENSOR PX100.64.160.02 (XSENSOR Technology Corporation, Calgary, Canada) pressure mapping system with its X3 software (v6) was used. The XSENSOR is considered to be the gold standard for pressure mapping and it has previously been used in several studies [33, 40–42]. The XSENSOR mat, fitted with over 10,000 sensing points, had a total area of 68.5 cm × 68.5 cm and a sensing area of 50.8 cm × 50.8 cm, resulting in approximately 200 cells per area [43]. Manufacturer's specification also indicated that the pressure mat had low hysteresis and low creep, an accuracy rate of ±10% of the calibrated values, sampling frame rate of 15.8 per second, and a spatial resolution of 0.51 cm. IP readings were transmitted from the XSENSOR mat to a hand-held monitor. The pressure mapping was conducted on an Arco TN 0055 X-ray table to mimic the imaging surfaces used in radiotherapy. The X-ray table was 240 cm long, 85.3 cm wide, and 2.2 cm thick, made from an industrial grade Rohacell carbon fibre with 0.9 mm aluminium (Al).

### 2.6. Procedure for Pressure Mapping

The mat was fixed securely to the X-ray table with adhesive tape. To standardise volunteer positioning, a measurement of 2 cm from top (head) of the mat was taken and a tape was placed there to ensure that all volunteers had their heads placed on the same point of the mat. Volunteers were asked to lie on the mat in a supine position with the hands pronated and the hips adjusted to ensure that they were equidistant from the edges of the mat. Following a six-minute settling time [33, 44], the volunteers were asked to remain still for two minutes, whilst pressure mapping data were acquired to serve as control data. At the end of the two minutes, the volunteer was helped off the X-ray table, the silicone gel surface overlay was placed under the pressure mat, and the volunteer was asked to lie again on the pressure mat for the intervention pressure mapping data to be acquired. The same settling and pressure mapping data acquisition times (six and two minutes respectively) were used for the intervention data collection. During pressure mapping, access to the imaging room was restricted to protect volunteers' privacy and also to avoid any distraction. The data acquired were saved onto the hand-held Xsensor device. To ensure high levels of infection control and hygiene, the pressure mat was cleaned in between volunteers using wet wipes as recommended by the manufacturer.

## 3. Results

### 3.1. Demographics

The sample comprised 14 males (70%) and six females (30%), with an age range of 25–53 years (mean = 34.4 ± 7.0).

### 3.2. Normality Testing

The results of normality testing (Kolmogorov–Smirnov tests, *p* ≥ 0.05) indicated that the data were normally distributed for all the four variables (mean IP for the head on the X-ray table with and without the silicone gel surface overlay, and the mean peak pressure index (PPI) for the head on the table with and without the gel surface overlay, in mmHg). The mean IP corresponds to the mean of all the cells in the contact area of the head. The mean PPI is defined as the mean of the highest IP within a 10–12 cm^2^ area per each frame [42, 45]. In other words, the mean PPI corresponds to the mean of the maximal values within the area under investigation. Studies have shown that this area (10–12 cm^2^) is equivalent to a 3 × 3 cell matrix when using the Xsensor pressure mat [42, 45].

### 3.3. Inferential Statistics

Paired-samples *t*-test results indicated that there was a statistically significant decrease in the mean IP for the head on the X-ray table without the silicone gel surface overlay (mean = 83.9 ± 8.2 in mmHg) and the X-ray table with the gel surface overlay (mean = 62.4 ± 6.1 in mmHg), *t* (19) = 14.5, and *p* ≤ 0.001 (two-tailed). The mean decrease in IP was 21.5 mmHg with a 95% confidence interval ranging from 18.4 to 24.6. The eta squared statistic (0.9) indicated a large effect size. The given range of the mean IP values corresponds to the standard deviation of the mean.

**Figure 1:**
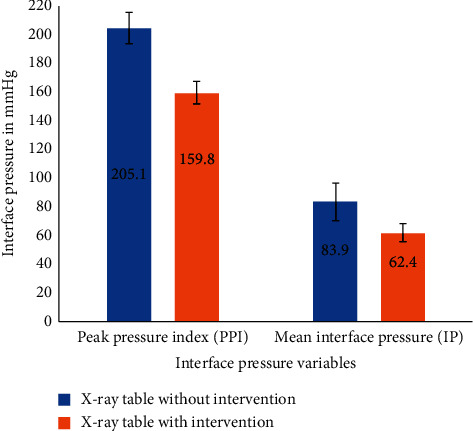
Comparison of mean IP and PPI with standard deviation on the X-ray table with and without the intervention.

Paired-samples *t*-test results indicated that there was a statistically significant decrease in the mean PPI for the head on the X-ray table without the silicone gel surface overlay (mean = 205.1 ± 28.2 in mmHg) and the X-ray table with the gel surface overlay (mean = 159.8 ± 26.8 in mmHg), *t* (19) = 5.5, and *p* ≤ 0.001 (two-tailed). The mean decrease in PPI was 45.3 mmHg with a 95% confidence interval ranging from 28.1 to 62.5. The eta squared statistic (0.6) indicated a large effect size. The given range of the mean PPI values corresponds to the standard deviation of the mean. The comparisons of mean IP and PPI on the X-ray table with and without the silicone gel surface overlay are presented graphically in Figure 1.

## 4. Discussion

This intervention study was conducted to assess the impact on IP values for the head through the use of a thin silicone gel surface overlay during radiographic procedures.

Findings showed that its use resulted in a statistically significant decrease in the mean IP for the head on the X-ray table without the silicone gel surface overlay (mean = 83.9 ± 8.2 in mmHg) and the X-ray table with the gel surface overlay (mean = 62.4 ± 6.1 in mmHg), *p* ≤ 0.001. Similarly, the use of the thin silicone gel surface overlay resulted in a statistically significant decrease in the mean PPI for the head on the X-ray table without the silicone gel surface overlay (mean = 205.1 ± 28.2 in mmHg) and the X-ray table with the gel surface overlay (mean = 159.8 ± 26.8 in mmHg), *p* ≤ 0.001. The findings of this intervention study can be compared to previous studies [46–50] which indicated that gel surface overlays have the ability to significantly reduce interface pressure risk thereby minimising the risk of PUs. It must be stated though that some of these studies were conducted on surfaces and subjects that were different from the conditions and subjects of this study. The silicone gel surface overlay used as an intervention in this study had viscoelastic properties, enabling it to support body weight and shift IP to a larger contact area without bottoming out. The movement of IP to a larger contact area significantly reduces the IP brought to bear on the head. Silicone gel surface overlays also have the ability to resist applied pressure and return to their original state when the applied pressure is removed [49]. These two properties, high viscosity and elasticity, are vital to ensure that the intervention can be applied during radiography and radiotherapy planning and treatment procedures because they minimise movement and errors.

The findings of this intervention study could contribute to efforts to reduce PU risks for the head, particularly among vulnerable patients accessing prolonged radiography and radiotherapy planning and treatment procedures. Patients who are likely to access these procedures are usually older, of poorer health, and mostly suffering from chronic diseases such as cancer [34]. Their advanced age comes with a marked reduction in the collagen and elastin content in their skin which makes them highly prone to experiencing skin injuries. Furthermore, the conditions and the specific characteristics of prolonged interventional radiography procedures (e.g., cervical vertebroplasty) and radiotherapy treatment procedures such as cranial stereotactic radiotherapy are likely to expose patients to MDR PUs.

The clinical implication of this study is that patients undergoing prolonged radiography and radiotherapy procedures could be provided with a thin silicone gel surface overlay behind their head. The application of the gel could result in a significant reduction in the interface pressure for the head by approximately 25%. This could have beneficial impact on patient management. However, to ensure that the silicone gel intervention does not have a negative impact on the imaging or therapy procedure, the intervention must be assessed to ensure that it meets the specific conditions within these specialised settings. For example, any silicone gel intervention that would be applied in radiography and radiotherapy planning and treatment must be assessed for its impact on image quality, radiation attenuation, and its ability to prevent patient movement. This is essential because any intervention that produces artefacts will degrade the diagnostic quality of a radiographic image possibly leading to inaccurate diagnosis [17]. As in other cases, this risk should be mitigated against its potential benefit; the benefit in this situation surrounds minimising the risk of developing a PU.

To successfully apply the silicone gel intervention in radiotherapy treatment procedures, it ought to be applied during radiotherapy planning. This is crucial because the radiotherapy planning parameters must be the same as that for treatment [18]. Prior to radiotherapy treatment procedures, patients must undergo a planning scan in a computerised tomography (CT) or a positron emission tomography-computerised tomography (PET-CT) machine. If a gel intervention is to be used during treatment, it has to be applied during the course of the planning to ensure reproducibility of patient position as well as the position of the target tumour, internal organs, and structures. This will help to ensure that the target tumour is not missed during treatment thereby improving the accuracy of the treatment [18].

## 5. Conclusion

The use of a thin silicone gel surface overlay could reduce IP risk for the head by approximately 25%. The reduction in IP risk could have a significant impact on reducing the risk of developing a PU. To ensure maximum benefit, the silicone gel surface overlay must be assessed to meet the specific needs within radiography and radiotherapy planning and treatment settings.

## Figures and Tables

**Table 1:** Physical characteristics of the five static surface overlays.

Name	Image	Material	Dimension (*L* × *W* × *T* in cm)^*∗*^	Weight (kg)
Gel table/hip pad	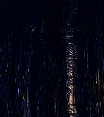	Gel	45 × 45 × 1.4	3.4
Silicone gel flat pad	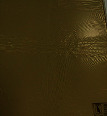	Silicone gel	45 × 45 × 0.7	1.4
Blue hollow overlay	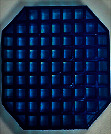	Elastic	6 × 6 × 1.6	<0.09
Sundance SUN Z3-S	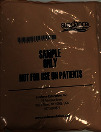	Fluidised	18 × 18 × 1.7	0.2
Small round gel	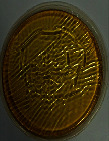	Gel	8.5 × 8.5 × 0.6	<0.09

*∗L* = length, *W* = width, and *T* = thickness.

**Table 2:** Mean recorded dose at low and high tube potentials with corresponding percentage changes.

Control (no overlay)	Mean dose for high kV (75 kV, 2 mAs)	37.3 mGy		
	Mean dose for low kV (50 kV, 2 mAs)	10.7 mGy		
Surface overlay	Mean dose at 75 kV, 2 mAs	Percentage decrease (%)	Mean dose at 50 kV, 2 mAs	Percentage decrease (%)

Gel table/hip pad	32.1 mGy	14.0	8.2 mGy	23.4
Silicone gel flat pad	34.2 mGy	8.3	9.6 mGy	10.3
Blue hollow overlay	35.1 mGy	5.9	10.3 mGy	3.7
Sundance SUN Z3-S	30.0 mGy	19.6	7.8 mGy	27.0
Small round gel	34.3 mGy	8.0	9.6 mGy	10.3

**Table 3:** Exposure and deviation indices for the surface overlays at high and low kVs.

	High kV (120, 1.2 mAs)	Low kV (60, 1.2 mAs)
Control	EI–2940.10	EI224.47
	TI–250	TI–250
	DI–10.70	DI–−0.46

Gel table/hip pad	EI–2705.38	EI–195.26
	TI 250	TI–250
	DI–10.34	DI–−1.07

Silicone gel flat pad	EI–2926.91	EI–213.16
	TI–250	TI–250
	DI–10.68	DI–−0.69

Blue hollow surface	EI–2842.58	EI–207.48
	TI–250	TI–250
	DI–10.55	DI–−0.80

Sundance SUN Z3-S	EI–2773.14	EI–197.91
	TI–250	TI–250
	DI–10.45	DI–−1.01

Small round gel	EI–3020.51	EI–222.46
	TI–250	TI–250
	DI–10.82	DI–−0.50

EI, TI, and DI all in units of microgray (*μ*Gy).

**Table 4:** Radiographic images of the five surfaces at high and low kVs.

Surface overlay	High kV	Low kV	High kV with hand	Low kV with hand	Artefact present
Gel table pad	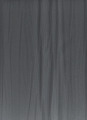	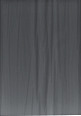	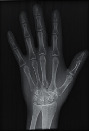	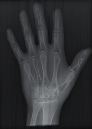	Yes
Silicone gel flat pad	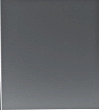	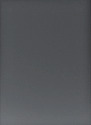	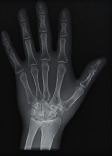	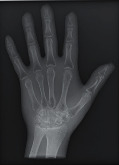	No
Blue hollow gel overlay	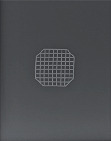	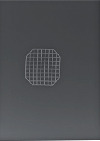	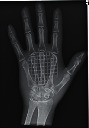	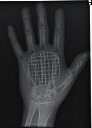	Yes
Sundance SUN Z3-S	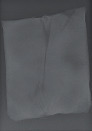	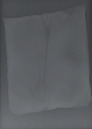	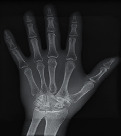	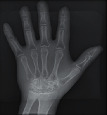	Yes
Small round gel	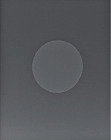	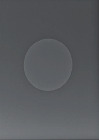	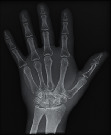	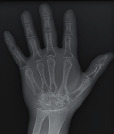	Yes

**Table 5:** The mean Hounsfield unit (HU) with standard deviation (SD) for the various surface overlays.

Surface no	Surface overlay	Mean HU ± SD
1	Gel table/hip pad	−0.67 ± 22.93
2	Silicone gel flat pad	−12.54 ± 26.80
3	Blue hollow surface	−508.96 ± 37.93
4	Sundance SUN Z3-S	−0.7 ± 15.3
5	Small round gel	−18.17 ± 7.06

## Data Availability

The data used to support the findings of this study are restricted by the University of Salford College of Health and Social Care Ethics Committee in order to protect volunteers' anonymity and confidentiality. The data are available from Seth Kwadjo Angmorterh and may be released upon application for researchers who meet the criteria for access to confidential data.
